# Avian Foxp3 acts as a switch for IL-10 by directly binding the T3G motif: revealing a novel epigenetic modulation paradigm

**DOI:** 10.1186/s12964-026-02967-0

**Published:** 2026-06-25

**Authors:** Tianxu Li, Shouwen Du, Yaxin Wu, Zhouying Chi, Wenjing Yang, Keyi Shi, Zhicheng Liu, Chunhong Zhang, Xian Zou, Mingfei Sun, Jianfeng Zhang, Ming Liao

**Affiliations:** 1 National and Regional Joint Engineering Laboratory for Medicament of Zoonosis Prevention and Control, Guangdong Provincial Key Laboratory of Zoonosis Prevention and Control, College of Veterinary Medicine, South China Agricultural University, Guangzhou, China; 2 State Key Laboratory of Swine and Poultry Breeding Industry, Key Laboratory for Prevention and Control of Avian Influenza and Other Major Poultry Diseases, Ministry of Agriculture and Rural Affairs, Key Laboratory of Livestock Disease Prevention of Guangdong Province, Institute of Animal Health, Guangdong Academy of Agricultural Sciences, Guangzhou, China; 3 State Key Laboratory of Swine and Poultry Breeding Industry & Guangdong Key Laboratory of Animal Breeding and Nutrition &, Institute of Animal Science, Guangdong Academy of Agricultural Sciences, Guangzhou, China; 4 College of Animal Science and Technology, Zhongkai University of Agriculture and Engineering, Guangzhou, China

**Keywords:** Immune regulation, Epigenetic activation, Chicken Foxp3, IL-10, H9N2 avian influenza virus

## Abstract

**Background:**

Foxp3 is the master transcription factor for regulatory T (Treg) cell differentiation and function, essential for immune homeostasis and tolerance. While its role is well-characterized in mammals, its functional mechanisms in avian species remain poorly understood.

**Methods:**

We conducted comparative genomic and functional analyses of chicken Foxp3 (chFoxp3). DNA-binding specificity was determined using motif analysis and genome-wide scanning. An H9N2 avian influenza challenge model was used to assess Foxp3 expression dynamics and CD4⁺CD25⁺ T cell frequency in vivo. The transcriptional regulation of IL-10 by chFoxp3 was investigated through promoter binding and transactivation assays, with key domains mapped via mutagenesis.

**Results:**

Avian Foxp3 has evolved independently from its mammalian ortholog. Unlike human Foxp3, which binds the FKHM motif, chFoxp3 specifically recognizes a T3G microsatellite motif. Genome-wide scanning identified potential targets enriched in the FoxO signaling pathway, including IL-10. In an H9N2 infection model, Foxp3 expression and CD4⁺CD25⁺ T cell frequency exhibited biphasic dynamics. Mechanistically, chFoxp3, but not human FoxP3, directly binds the T3G motif in the IL-10 promoter to activate transcription. This transactivation function critically depends on the HNT linker within its Fork-head domain.

**Conclusions:**

Our study reveals a novel mode of transcriptional regulation by chFoxp3 via T3G motif recognition, underscoring its functional diversification during evolution. These findings provide a foundation for understanding immune regulation in birds under both physiological and pathological conditions, such as avian influenza infection.

**Supplementary Information:**

The online version contains supplementary material available at https://doi.org/10.1186/s12964-026-02967-0.

## Introduction

The differentiation and development of regulatory T (Treg) cells are molecularly governed by the transcription factor fork-head box P3 (Foxp3), which not only determines lineage specification but also dynamically modulates suppressive capacity through epigenetic remodeling [1]. Structural analyses revealed that mammalian Foxp3 within multiple protein complexes interacts with RUNX1, Egr-1, and NFATC2 to repress proinflammatory cytokines (IL-2, IL-4, and IFN-γ) while inducing immunosuppressive mediators (IL-10, IL-35, and TGF-β) through both soluble factors and cell contact mechanisms [2–4]. Additionally, Foxp3 undergoes extensive posttranslational modifications (acetylation, ubiquitination) that fine-tune its stability and DNA-binding affinity, forming a tunable node for immune adaptation [5, 6]. The evolutionary conservation of Foxp3 across vertebrates, from teleost to primates, underscore its indispensable role in adaptive immunity [7, 8]. Chickens (*Gallus gallus*), a pivotal model in comparative immunology, possess functional Treg cells capable of suppressing T-cell proliferation in vitro [9]. During Marek's disease virus (MDV) infection, TGF-β^+^ Treg cells expand to establish immunosuppressive networks through sustained cytokine signaling [10]. Sustained immune tolerance in congenital avian leukosis virus subgroup J-infected chickens is maintained by the generation of high-frequency activated CD4⁺CD25⁺ T cell populations and the suppression of B cell function through the expression of the inhibitory cytokines TGF-β and CTLA-4 [11]. Genomic studies revealed that chicken Foxp3 (chFoxp3) shares 62% global amino acid identity with mouse Foxp3, retaining critical domains, including the fork-head DNA-binding motif and leucine zipper required for multimerization [12]. This structural conservation suggests that functional mechanisms may parallel those in mammalian systems, particularly in maintaining immune equilibrium.

Critical knowledge gaps persist in avian Treg biology. While mammalian Foxp3 interacts with DNA and other transcription factors to regulate Treg-specific gene expression, analogous epigenetic signatures in birds remain undefined [13, 14]. The impact of avian-specific pathogens (e.g., MDV and AIV) on Treg homeostasis is poorly understood, despite evidence that viral interference with Foxp3 exacerbates immunopathology in mammals [15, 16]. Commercial anti-Foxp3 antibodies fail to cross-react due to epitope divergence in avian species [12]. Addressing these limitations holds dual significance. From an evolutionary perspective, chickens provide insights into how Treg biology adapts to environmental pressures, since their accelerated lifespan and intense pathogen exposure may drive unique regulatory strategies [17]. Agriculturally, modulating Treg activity could mitigate economic losses from poultry respiratory/enteric diseases [18]. For example, Salmonella-infected chickens exhibit expanded Treg populations that impair Th1-mediated bacterial clearance, suggesting that transient Treg inhibition could enhance host defense [19]. Spatial distribution analyses revealed that avian CD4⁺CD25⁺ T cell cells are predominantly localized at mucosal interfaces, particularly in the intestinal lamina propria and bronchus-associated lymphoid tissue [9, 20]. Furthermore, the gut microbiota-Treg-Th17 axis critically influences growth performance-a metric that directly affects poultry industry profitability [21]. Despite these challenges, elucidating the regulatory mechanisms governing avian Treg cells is highly important for both immunological research and poultry industry applications.

H9N2 avian influenza virus (AIV), classified as a low-pathogenicity virus, has been reported to induce complex immune dysregulation in hosts [22]. Current evidence suggests that H9N2 AIV may interfere with host immunity through multiple molecular pathways. For example, the NS1 protein of H9N2 AIV has demonstrated the capacity to suppress interferon signaling pathways and modulate the expression of both antiviral and proinflammatory cytokines [23]. Another proposed mechanism involves virus-induced elevation of adrenal corticosteroids, which could enhance thymocyte apoptosis and compromise T-cell development [24]. Treg cells, a specialized CD4⁺T-cell subset, play a pivotal role in immune homeostasis by balancing pathogen clearance and autoimmunity prevention [24]. While H5N1 AIV infection has been shown to manipulate Treg populations to suppress CD8⁺T-cell responses via IL-10-dependent mechanisms, the specific immunological interplay between H9N2 AIV and Treg cells remains poorly characterized [25].

In the present study, we aimed to elucidate the molecular mechanism by which chFoxp3 orchestrates epigenetic reprogramming to increase IL-10 production, thereby providing novel insights into avian immune regulation during H9N2 AIV infection. Utilizing an established H9N2-infected chicken model, we first delineated the dynamic changes in chFoxp3 expression and CD4⁺CD25⁺ T cell frequency. Through integrated sequence analysis, DNA-binding profiling, and genome-wide target prediction, we identified the T3G motif as a key binding site for chFoxp3 and revealed its extensive regulatory network involving critical immune pathways, notably IL-10 signaling. Functional validation confirmed that chFoxp3 directly binds to T3G-containing promoters to transactivate IL-10 expression, highlighting an evolutionarily adapted strategy to fine-tune inflammatory responses. These findings not only bridge a fundamental knowledge gap in avian Treg biology but also offer strategic insights for the development of immunomodulatory interventions to increase poultry health and disease resistance.

## Materials and methods

### Virus and cells

A H9N2 AIV strain (A/Chicken/Guangdong/10142/2018) was maintained in our laboratory and propagated in 10-day-old specific-pathogen-free (SPF) chicken embryos. Virus titration was determined by using 50% egg infectious dose assay (EID_50_/ml) in SPF chicken embryos, and titers were calculated using the Reed and Muench method. HEK293T/17 (CRL-11268) cells were purchased from ATCC. UMNSAH/DF-1 (GNO30) cells were purchased from the Cell Bank of the Chinese Academy of Sciences. All the cells were cultured in high-glucose DMEM (C11995500BT, Gibco) supplemented with 10% fetal bovine serum (A5256701, Gibco) and antibiotics.

### Animal experiments

All the chicken experiments were conducted with four-week-old SPF chickens purchased from Boehringer Ingelheim Viton Biotechnology Co., Ltd. (Beijing, China). H9N2 AIV was administered to chickens via intranasal instillation of 10^7^ EID₅₀ per bird. The chickens were housed in individual cages within a temperature-controlled environment (28°C-30°C) with 60%−70% humidity and were provided with standard feed and water ad libitum. Oropharyngeal swabs were collected daily for the detection of virus shedding. At various time points, the blood samples were collected.

### Flow cytometry

The cells were washed and prepared as single-cell suspensions in FCM staining/wash buffer (2% BSA, 25 mM HEPES, and 2 mM EDTA in PBS) and stained for 30 min at 4°C with antibody cocktails, including mouse anti-chicken CD3-APC (8200–11, Southern Biotech), mouse anti-chicken CD4-PE (8255–09, Southern Biotech), and mouse anti-chicken CD25-FITC (HCA173, Bio-Rad). The stained cells were analyzed with CytoFLEX (Beckman Coulter). The FACS data were analyzed with FlowJo 10.6.2 software (Tree Star).

### Quantitative real-time PCR

Total RNA was extracted with a FastPure Cell/Tissue Total RNA Isolation Kit (RC112-01, Vazyme). The obtained RNA was reverse-transcribed into cDNA via Hifair III 1 st Strand cDNA Synthesis SuperMix for qPCR (gDNA digester plus) (11141ES60, Yeasen). qRT-PCR was performed via the ArtiCan SYBR qPCR Mix (TSE401, TSINGKE) on a LightCycler 96 instrument (Roche). The relative mRNA expression level was calculated via the 2^^−(∆∆Ct)^ method [26]. The primer sequences are listed in Table S1.

### Cloning of genes

Genomic DNA was extracted via the TIANamp Genomic DNA Kit (DP304, Tiangen). The extracted genomic DNA and cDNA were used as templates, and PCR mix (PK511, Vazyme) was used to amplify the gene CDs or promoter region DNA sequences. The primer sequences are listed in Table S1.

### Construction of plasmids

The DNA sequence encoding chFoxp3 was chemically synthesized via codon optimization for expression in *E. coli* and subsequently cloned and inserted into the expression vector pET28a (+) between the NcoI and XhoI restriction sites (TsingKe). The full-length coding sequence (CD) regions of the chicken and human Foxp3 genes were subsequently cloned and inserted into the eukaryotic expression vector pcDNA3.1-Flag-HA via the BamHI and EcoRI restriction sites. Additionally, the promoter regions of chicken IL-10 and human IL-10 were inserted into the reporter vector pGL6-TA (D2105, Beyotime) between the NheI and HindIII restriction sites. The sgDNA sequences were cloned at the BbsI site into pLentiCRISPR v2-cmv-mCherry-GSGP2A-puro (Table S2). The helper plasmids pMD2.G and psPAX2, were obtained from the MiaoLing Plasmid Sharing Platform (Wuhan, China). Site-directed mutagenesis of the plasmids was performed via the Hieff Mut Site-Directed Mutagenesis Kit (11003ES10, Yeasen). All the recombinant plasmids were constructed via seamless cloning via the Hieff Clone Plus One Step Cloning Kit (10,911, Yeasen). Finally, the integrity of all the plasmids was confirmed by sequencing.

### Lentiviral infection and promoter motif knockout

The lentiviruses were produced by co-transfecting plasmids into HEK293T/17 cells using PEI (HY-K2014, MCE). For infection, DF-1 cells were transduced with the viral particles in the presence of 8 μg/ml polybrene (40804ES86, Yeasen). The transduced cells were then selected using 10 μg/ml puromycin (ant-pr-1, Invivogen). The bulk cell population was transfected with single-stranded DNA (ssDNA) donor (Table S2) using Lipofectamine 3000 (L3000015, Invitrogen). The precise knockout efficiency at the promoter motif was determined by T7EI cleavage assay (D7080, Beyotime) and sequencing.

### Expression and purification of the recombinant chFoxp3 protein

The recombinant plasmid pET-28a-chFoxp3 was transformed into *E. coli* BL21 (DE3) cells for protein expression. The transformed cells were grown at 37°C in LB media supplemented with 50 μg/ml kanamycin until the OD_600_ reached 0.6. Protein expression was induced with 0.5 mM IPTG, and the cells were further incu-bated at 25°C for 24h. The cells were harvested by centrifugation and then resuspended in TieChui Lysis Buffer (BR0005, ACE) at 4°C for 30 min. The lysate was then centrifuged at 8,000 × g for 30 min at 4°C to separate the supernatant and inclusion bodies, which were subsequently analyzed by SDS-PAGE. The recombinant protein was purified using a High Affinity Ni-charged Resin FF (L00666, Genscript) according to the manufacturer’s instructions. Recombinant mouse Foxp3 protein (Tyr190-Glu412) (RPB877Mu02) was purchased from CLOUD-CLONE CO., LTD (USA).

### Size exclusion chromatography analysis

The oligomeric state of recombinant proteins was analyzed by Tsingke Biological Co., Ltd (Nanjing, China). This analysis utilized a high-performance liquid chromatography system (ZHEJIANG FULI ANALYTICAL INSTRUMENTS CO., LTD). A Sepax SRT SEC-300 size exclusion column was employed, with PBS as the mobile phase and a flow rate of 1.0 ml/min. Detection was carried out using a UV–visible detector (UVD) at a wavelength of 280 nm. the chromatography run time was 20 min. Data analysis was based on the elution curves of standard proteins.

### Circular dichroism spectroscopy

Protein secondary structure analysis was performed using a circular dichroism spectropolarimeter (e.g., Jasco J-1500). Purified chFoxp3 was exchanged into a low-UV-absorption buffer (5 mM sodium phosphate buffer, pH 7.4), and the concentration was adjusted to 0.15 mg/ml. Spectra were acquired in the far-UV region (190–260 nm) using a 0.1 cm path length quartz cuvette, with a scanning speed of 50 nm/min, response time of 1 s, and bandwidth of 1 nm. Each spectrum represents the average of 3 accumulations, with buffer background subtracted. To assess thermal stability, the ellipticity at 222 nm was monitored as a function of temperature (20-90°C with a ramp rate of 1°C/min), and the melting temperature (Tm) was determined from the resulting denaturation curve.

### SDS-PAGE and western blot

Protein samples were prepared using SDS-PAGE Protein Loading Buffer (AIWB-002, Affinibody) and denatured by heating at 100°C for 10 min. The denatured proteins were separated via electrophoresis on 10% SDS-PAGE gels at a constant voltage of 200 V for 1 h. After electrophoresis, the gels were stained with Coomassie Brilliant Blue (G2059, Servicebio) for protein visualization. For Western blot analysis, proteins were transferred to 0.22 µm nitrocellulose membranes (10,600,001, Cytiva) using the eBlot L1 Fast Wet Transfer System (Genscript). The membranes were blocked with protein-free rapid blocking solution (G2052, Servicebio) for 10 min at room temperature and then incubated overnight at 4°C with the following primary antibodies: mouse anti-His monoclonal antibody (1:5,000; 66,005–1-Ig, Proteintech), mouse monoclonal anti-Flag antibody (1:2,000; F1804, Sigma-Aldrich), or Recombinant Rabbit anti-β-Actin Antibody (1:5,000; GB15003, Servicebio). The membranes were subsequently incubated for 1 h at room temperature with the following secondary antibodies: DyLight 680-conjugated Goat anti-Mouse IgG (H + L) (1:10,000; 35,518, Thermo Fisher), or DyLight 800-conjugated Goat anti-Rabbit IgG (H + L) (1:10,000; SA5-10,036, Thermo Fisher). The protein bands were visualized and imaged with a Sapphire FL Imaging System (Azure Biosystems, Dublin, CA).

### Immunofluorescence and confocal microscopy

The cells cultured on glass-bottom plates were fixed with 4% paraformaldehyde for 10 min at room temperature, permeabilized with Enhanced Immunostaining Permeabilization Buffer (P0097, Beyotime) for 5 min at room temperature, and blocked with protein-free rapid blocking solution (G2052; Servicebio) for 10 min at room temperature. The cells were subsequently incubated with mouse anti-Flag antibodies (F1804, Sigma) (1:1,000) overnight at 4°C, followed by incubation with Cy3-conjugated Goat Anti-Mouse IgG (H + L) (SA00009-1, Proteintech; 1:500) for 1 h at room temperature. Nuclei were counterstained with DAPI, and images were acquired via a Zeiss LSM 900 laser scanning confocal microscope.

### DNA design and synthesis

Double-stranded (ds) DNA was annealed from single-stranded (ss) complementary oligos. HPLC-purified 5' biotin-labeled or unlabeled ssDNA was synthesized by TsingKe Biotechnology Co., Ltd. (Table S3). After each oligonucleotide pellet was briefly centrifuged, the ssDNA was dissolved in Annealing Buffer for DNA Oligos (D0251, Beyotime). The complementary ssDNA was then mixed in equimolar amounts, heated to 95°C for 2 min, and then subjected to a temperature decrease program of 0.1°C per 8 s until it cooled to 25°C.

### Electrophoretic mobility shift assay

EMSAs were performed via a LightShift Chemiluminescent EMSA Kit (20,148, Thermo Scientific). Briefly, purified chFoxp3 protein (2 μg) and DNA probes (0.02 μm) were incubated for 30 min at room temperature and analyzed on a 5% TBE PAGE gel (D0168S, Beyotime). The complexes were transferred to nitrocellulose membranes (HATF00010, Millipore). Biotin-labeled DNA was detected by chemiluminescence and captured with a Tanon 5200 chemiluminescence imaging system.

### Enzyme linked immunosorbent assay

Chicken sera were subjected to ELISA using a chicken IL-10 ELISA kit (ml059830, mlbio) according to the manufacturer’s instructions. The absorbance at 450 nm was measured via a Spark multimode plate reader (Tecan). Assays were performed in duplicate, and the data are expressed as the means ± SEMs.

### Luciferase reporter assay

Cells were grown to 70% confluence in 96-well white opaque plates (FCP968, Beyotime) and co-transfected with reporter plasmids along with either expression or empty control plasmids via Lipofectamine 3000. After 36 h, firefly luciferase activity was measured via the One-Lumi II Firefly Luciferase Reporter Gene Assay Kit (RG056, Beyotime) following the manufacturer’s protocol. Briefly, 100 µL of luciferase assay buffer containing firefly luciferase substrate was added to each well and incubated for 5 min at room temperature. Luminescence was quantified via a Spark multimode microplate reader (Tecan).

### Chromatin immunoprecipitation assay

The cells were washed with PBS and subsequently crosslinked with 1% formaldehyde (F809702, Macklin) for 10 min. The reaction was quenched with 125 mM glycine (A502065, Sangon Biotech) for 5 min. ChIP was performed via the ChainFree Flag-Tag ChIP Kit (FI8904, FITGENE). Briefly, the cell pellets were lysed with lysis buffer containing a protease inhibitor cocktail (P001, NCM Biotech). Chromatin was sheared under optimized sonication conditions. The sheared chromatin was immunoprecipitated with ChainFree anti-Flag magnetic beads. Prior to immunoprecipitation, 1% of the sheared chromatin from each treatment group was reserved as input. The complexes were incubated with ChainFree anti-Flag magnetic beads at 4°C for 4 h. The beads were then washed, and the protein-DNA complexes were eluted via reverse crosslinking at 65°C for 6 h. The samples were subsequently treated with RNase A at 37°C for 1 h, followed by proteinase K digestion at 55°C for 2 h. Finally, the DNA was purified via phenol-chloroform extraction and precipitated with ethanol.

### Software and bioinformatics

The Foxp3 sequences were aligned via MEGA 12 via the neighbor‒joining method [27]. To evaluate the reliability of the phylogenetic relationships, a bootstrap analysis with 1000 replicates was performed. The resulting phylogenetic tree was visualized via Evolview (version 3) [28]. The prediction of protein domains, secondary structures, hydrophobicity, and disordered regions was performed via InterPro, AlphaFold3, ProtScale, and AIUPred, respectively [29–32]. The three-dimensional structure of the proteins was predicted via AlphaFold3. The results were visualized in PyMOL (version 3.0.3) (PyMOL Molecular Graphics System, Schrödinger). A custom Python script was used to align the motif to the chicken genome (Ensembl release 106: GRCg7b) within the promoter region of each gene (defined as the 2,000 bp upstream of the coding region) [33]. T3G (TGTTTGT) density distribution analysis was conducted via the CMplot package in R (version 4.4), which employs a 1 Mb sliding window for motif density calculation. The genome was partitioned into consecutive fixed-size intervals designated bins, followed by quantification of motif counts within each bin to compute density values. KEGG pathway annotation was performed, and the results were visualized with the OmicShare tool [34]. The Sankey diagram was generated with the ggsankey package in R. The exon structure of the gene was visualized via the Gene Structure Display Server 2.0 software. All the logo pictures were generated via WebLogo (version 3.3).

### Statistical analysis

The quantitative data are presented as the means ± SEMs. All experiments were repeated as at least three biological replicates, and each biological replicate was carried out with three technical triplicates. Statistical analyses were conducted via Prism 10 software (GraphPad Software, Inc.). Statistical comparisons between groups were analyzed for significance via one-way analysis of variance (ANOVA) and Student’s *t* test. Probability values of *P* < 0.05 were considered statistically significant. *, 0.01 < *P* < 0.05; **, 0.001 < *P* ≤ 0.01; ***, *P* ≤ 0.001.

## Results

### Evolutionary divergence in the genetic and structural landscape of avian and mammalian Foxp3 proteins

**Fig. 1:**
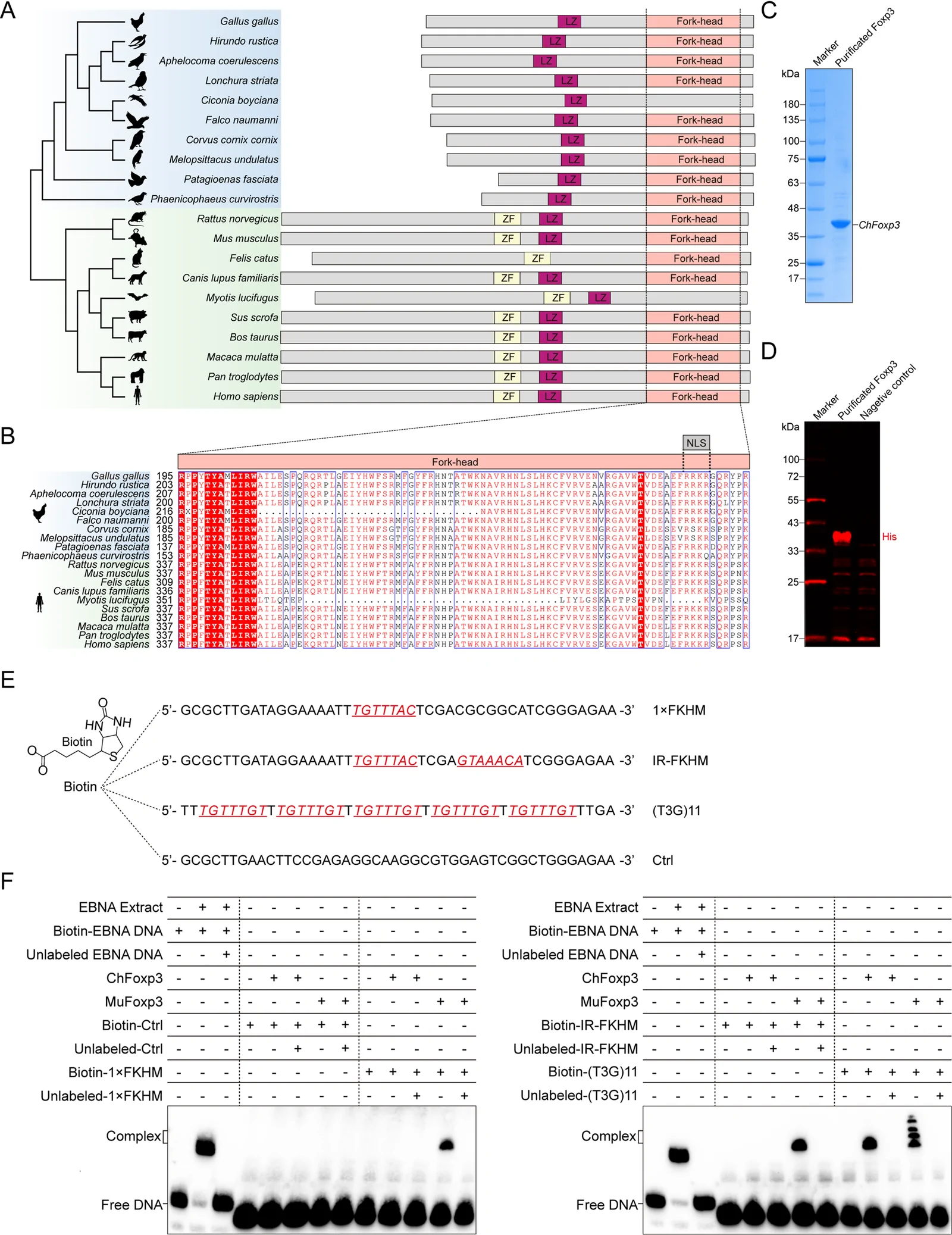
ChFoxp3 recognizes T3G microsatellites. A Phylogenetic analysis and domain architecture of Foxp3. ZF, zinc finger domain; LZ, leucine zipper domain; Fork-head, Fork-head domain. B Alignment of fork-head domain of Foxp3. Fully conserved amino acids are highlighted in shaded red, whereas partially conserved amino acids are enclosed within blue boxes. NLS, nuclear localization signal. C SDS-PAGE analysis of purified chFoxp3. D Western Blot validation of purified chFoxp3. E Schematic diagram of the 5'-biotin-labeled DNA probe sequence for 1×FKHM, IR-FKHM, (T3G)11, and Ctrl. F EMSA analysis of purified chFoxp3 and muFoxp3 protein (2 μg) with DNA probes (0.2 μM). A 100-fold excess of unlabeled DNA was added as a competitive control. Lanes 1-3, the control Epstein-Barr virus nuclear antigen (EBNA) system results

To gain deeper insight into the functional mechanisms of avian Foxp3, we cloned and identified the coding sequence of the chFoxp3 gene (Fig. S1A) and systematically analyzed its protein structural characteristics. Phylogenetic tree analysis revealed that avian Foxp3 has lower homology with mammalian Foxp3, which resides in different evolutionary branches (Fig. 1A). Conserved domain analysis revealed that the fork-head domain is highly conserved among Foxp3 proteins across various species, with both avian and mammalian cells having similar N-terminal proline-rich disordered sequences, coiled-coil domains, leucine zippers, and C-terminal nuclear localization sequences (Fig. 1A-B, Fig. S1B). However, there are also significant differences in Foxp3 expression between avian and mammalian species. Avian Foxp3 lacks several features present in mammalian Foxp3, including an N-terminal nuclear export signal, LxxLL motif, zinc finger domain, and RUNX1 binding domain. Sequence analysis revealed that human Foxp3 (huFoxp3) has numerous posttranslational modification sites, including phosphorylation, methylation, acetylation, ubiquitination, and glycosylation sites (Fig. S1B). In contrast, avian Foxp3 lacks many of these modification sites compared with mammalian Foxp3. These data demonstrate that the Foxp3 gene has experienced divergent evolutionary paths in avian and mammalian lineages, undergoing independent, lineage-specific evolution under distinct selection pressures, such that each has evolved distinct downstream regulatory networks and molecular mechanisms.

### ChFoxp3 specifically recognizes the T3G motif but not the FKHM motif

To compare the structural differences between chFoxp3 and its mammalian counterparts, we performed structural modeling of chFoxp3 and mouse Foxp3 via AlphaFold3. Although the overall architecture of these two proteins differs significantly, their functional fork-head domains (DNA-binding domains) demonstrate highly conserved structural configurations (Fig. S2). These interspecies structural differences provide a crucial molecular foundation for further elucidating the specific immunomodulatory network of avian Foxp3. Despite the presence of a Fork-head domain in chFoxp3, similar to that in other species, which is known to mediate DNA binding [35], the exact mechanism of its functionality remains unclear. To investigate the DNA-binding motif characteristics of avian Foxp3 via pull-down, we employed a prokaryotic expression system to express and purify the 6 × His-tagged chFoxp3 protein. However, the ProtScale (Fig. S3A) and AIUPred (Fig. S3B) analyses revealed that chFoxp3 shares moderate hydrophobicity and a high degree of disorder in its N-terminus (1–184 aa) with human or mouse Foxp3 [36, 37], which may hinder its soluble expression [29]. Consequently, chFoxp3 was expressed in inclusion bodies, and its expression optimization under temperature gradients (15°C-37°C) resulted in a maximal yield at 25°C (Fig. S3C). Subsequently, purification was performed via imidazole gradient elution (20–500 mM), and 500 mM imidazole effectively eluted the protein (Fig. S3D). The refolding conditions were systematically refined, yielding chFoxp3 with > 90% purity (Fig. 1C-D). Native PAGE and HPLC results showed that the recombinant chFoxp3 protein existed in both monomeric and dimeric forms (Fig. S3E-H). This finding suggests that chFoxp3, like its mammalian homologs, has the potential to form homodimers. Circular dichroism analysis indicated that the recombinant chFoxp3 protein was partially successfully refolded (Fig. S3I).

Next, to investigate whether chFoxp3 can bind known mammalian Foxp3 motifs, including the FKHM motif (TGTTTAC) and the T3G motif (TGTTTGT) [37, 38], we synthesized 5'-biotin-labeled or unlabeled DNA probes corresponding to the above motifs and performed EMSA. As a positive control, mouse Foxp3 bound to the 1 × FKHM and IR-FKHM motifs, as previously reported. Moreover, mouse Foxp3 typically binds T3G as a multimer, producing a characteristic ladder pattern. In contrast, chFoxp3 did not bind either the 1 × FKHM or IR-FKHM motifs. Although chFoxp3 bound to the T3G repeat sequence, it did not produce the characteristic ladder pattern bands observed for mouse Foxp3, indicating a marked difference in DNA-binding properties between chFoxp3 and mouse Foxp3. These findings suggest that chFoxp3 may lack the ability of mammalian Foxp3 to bind T3G in a multimeric form (Fig. 1E-F).

### Landscape and functional characterization of potential chFoxp3 target genes driven by binding to the T3G motif

**Fig. 2:**
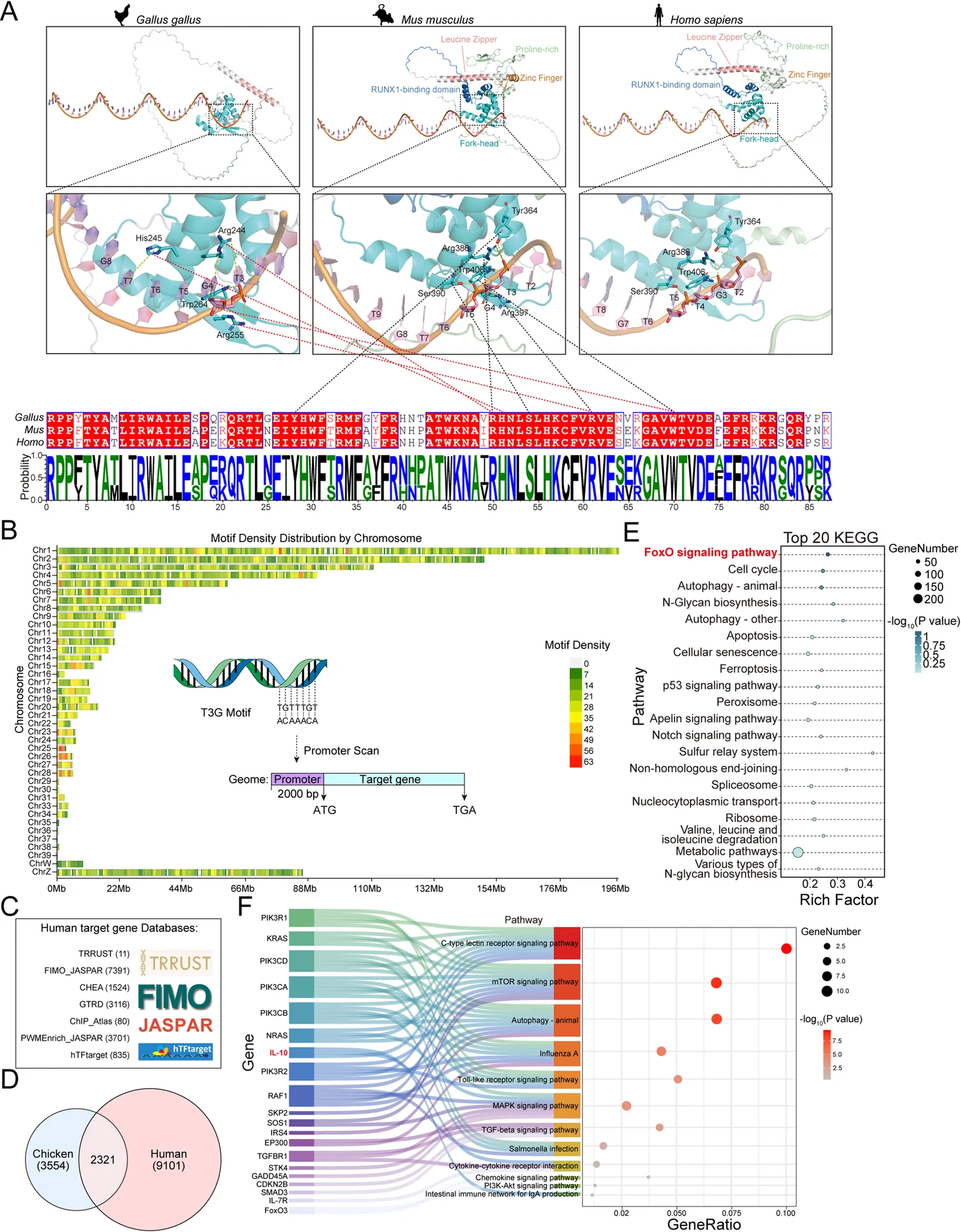
ChFoxp3 exerts immunoregulatory effects by binding to the T3G motif. **A** Structural representation of Foxp3 (*Gallus gallus, Mus musculus,* and *Homo sapiens*) binding to T3G microsatellites. **B** Chromosomal distribution of T3G motif density and a schematic representation of T3G motif retrieval in the chicken genome. **C** Schematic representation of human Foxp3 target gene retrieval via transcription factor databases (TRRUST, FIMO_JASPAR, CHEA, GTRD, ChIP_Atlas, PWMEnrich_JASPAR, and hTFtarget). **D** Venn diagram of huFoxp3 and chFoxp3 target genes. **E** Top 20 significantly enriched KEGG pathways for chFoxp3 target genes. **F** Sankey diagram depicting the KEGG enrichment results of chFoxp3 target genes in the FoxO signaling pathway

By separately predicting the structures of chicken, mouse, and human Foxp3 binding to the T3G motif, simulated structural diagrams and predicted binding sites were generated. The structural modeling results showed that the binding mode of mouse and human Foxp3 to the T3G motif was essentially identical, whereas the binding mode of chFoxp3 differed substantially from that of mammalian Foxp3. Mouse and human Foxp3 bind the T3G motif through Tyr364, Arg386, Ser390, Arg397, and Trp406, while chFoxp3 binds via Arg244, His245, Arg255, and Trp264, reflecting interspecies differences in Foxp3 function (Fig. 2A).

To determine whether the T3G motif is present in the chicken genome, we initially performed a genome-wide scan via a custom Python script. The results revealed that the T3G motif is enriched in promoter regions of multiple genes distributed across all chicken chromosomes (Chrs) except Chr32. Strikingly, the T3G motif was highly concentrated on Chr25 and Chr26, two relatively short chromosomes (Fig. 2B) (Table S4). However, incomplete genome annotation in chickens results in a significant proportion of these genes lacking functional annotations [39]. To assess the evolutionary conservation of transcriptional regulation, we performed cross-species analysis comparing mammalian Foxp3 target genes (curated from public databases) with T3G-responsive genes in chicken genomes. Given the paucity of high-confidence murine Foxp3 targets, we utilized the better-characterized huFoxp3 regulatory network as the primary comparator for interspecies functional alignment. For comparative analysis, the huFoxP3 target genes were systematically curated from seven independent databases: TRRUST (11 genes), FIMO_ASPAR (7,391 genes), CHEA (1,524 genes), GTRD (3,116 genes), ChIP_Atlas (80 genes), PWMEnrich_JASPAR (3,701 genes), and hTFtarget (835 genes) (Fig. 2C) (Table S5). After eliminating interdatabase redundancies and intersecting the remaining entries with chicken T3G-associated genes, we identified 2,231 conserved orthologous targets (Fig. 2D) (Table S6).

We next performed KEGG and GO enrichment analyses on the protein products of these potential chFoxp3 target genes. KEGG pathway analysis demonstrated that these overlapping genes are enriched primarily in environmental information processing, cellular processes, metabolism, genetic information processing, organismal systems, and human disease pathways (Fig. S4A) (Table S7). A significant number of genes were enriched in the "signal transduction" and "signaling molecules and interaction" pathways, which are closely associated with cellular signaling, suggesting a potential regulatory role of chFoxp3 in this process. By ranking the enriched KEGG pathways on the basis of their rich factor values (adjusted P values), we identified the FoxO pathway as the top-ranked pathway (Fig. 2E). Both the FoxO and Foxp families belong to the fork-head superfamily, which is characterized by a conserved fork-head DNA-binding domain [35, 40]. The genes associated with the FoxO pathway were categorized according to their functional roles, which revealed their involvement in several signaling pathways, including the C-type lectin receptor signaling pathway, MAPK signaling pathway, TGF-β signaling pathway, cytokine‒receptor interaction and influenza virus infection (Fig. 2F). Extensive cross-talk was observed among these genes, most of which are implicated in immunomodulatory functions. GO analysis revealed significant enrichment of these genes in pathways related to cellular processes, metabolic processes, biological regulation, and the regulation of biological processes (Fig. S4B, C) (Table S5).

### Foxp3 orchestrates immunosuppression and its temporal dynamics during AIV infection

**Fig. 3:**
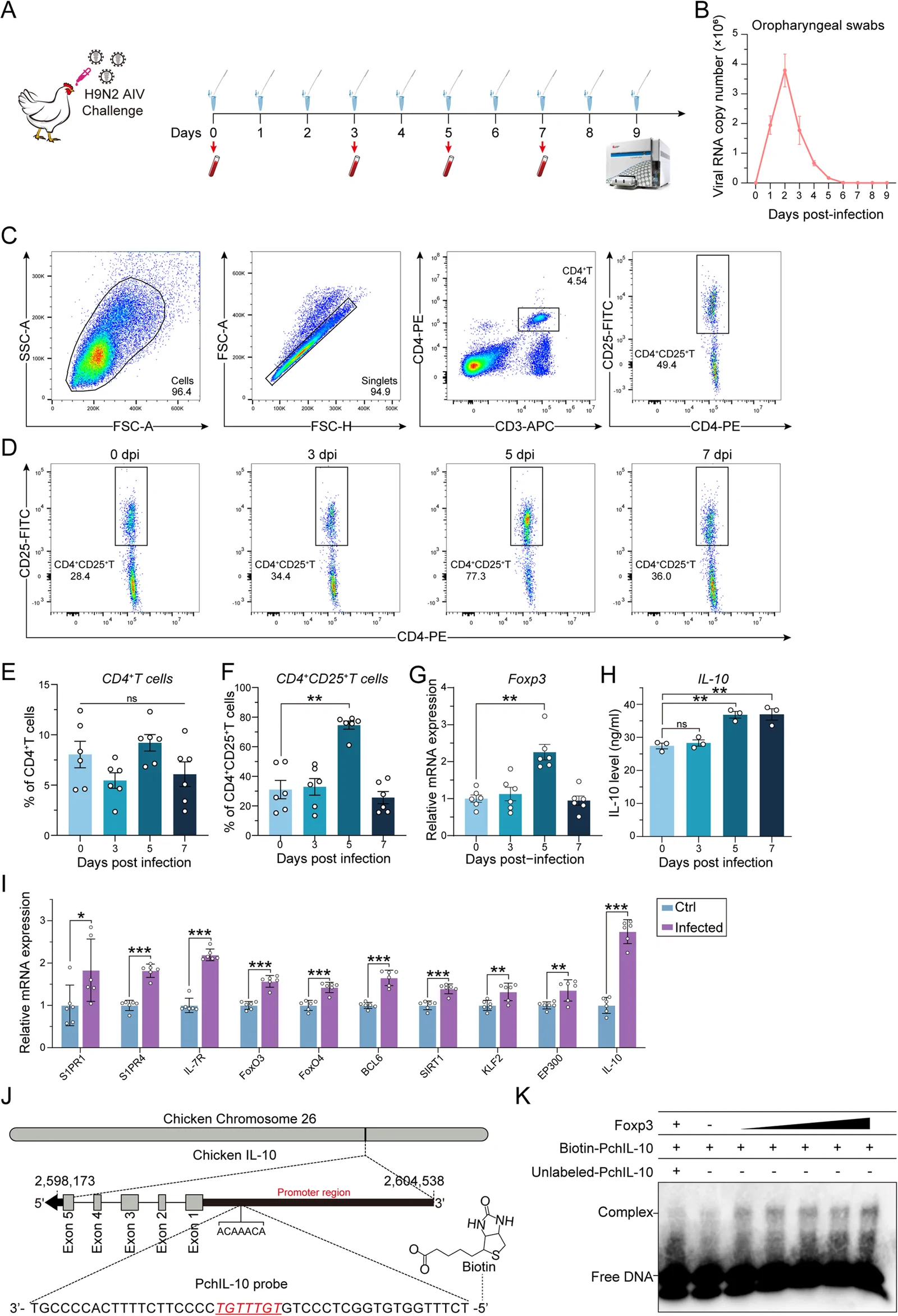
ChFoxp3 is involved in the immune response of chickens infected with H9N2 AIV. **A** Schematic representation of the experimental design and sample collection timeline following H9N2 AIV challenge in chickens. **B** Viral titers in oropharyngeal swabs (n = 6). **C** Gating strategy for flow cytometry analysis in chicken PBMCs.** D** Representative flow cytometry density plots showing temporal changes in CD4^+^ CD25^+^ T cell populations at selected time points (0, 3, 5, and 7 dpi) (n = 6). **E–F** Quantitative analysis of CD4^+^ T cell and CD4^+^ CD25^+^ T cell population frequencies (n = 6). *, 0.01 < *P* < 0.05; **, 0.001 < *P* ≤ 0.01; ***, *P* ≤ 0.001.** G** Relative mRNA expression of chFoxp3 in PBMCs (0, 3, 5, and 7 dpi) (n = 6).** H** IL-10 levels in the serum (n = 3). **I** Relative mRNA expression of immune-related chFoxp3 target genes in PBMCs at 5 dpi (FoxO3, FoxO4, S1PR1, S1PR4, EP300, BCL6, IL-7R, IL-10, SIRT1, and KLF2) (n = 6). **J** Schematic diagram of the chromosomal localization and gene structure of the promoter regions of the chicken IL-10 gene and the 5'-biotin-labeled DNA probe sequence for PchIL-10. **K** EMSA analysis of purified chFoxp3 (0–5 μg) protein with PchIL-10 probes (0.2 μM). A 100-fold excess of unlabeled DNA was added as a competitive control

To investigate the temporal dynamics and mechanistic basis of Foxp3 and CD4^+^CD25^+^ T cell regulation in infectious disease progression, we employed a chicken model with low-pathogenicity H9N2 AIV (Fig. 3A). The single H9N2 AIV infection model revealed that peak respiratory viral shedding occurred between 2 and 3 dpi, which was followed by a progressive decline to undetectable levels by 5 dpi (Fig. 3B), reflecting the hallmark progression of low-pathogenicity AIV infections [41]. The results indicated that the expansion of CD4⁺CD25⁺ T cells at 5 dpi, which increased to 77% of the population significantly higher than pre-infection levels (Fig. 3C-F). This elevated CD4⁺CD25⁺ T cell proportion returned to baseline by 7 dpi. This dynamic progression suggests the initiation of a highly coordinated immune response and regulatory program following infection. To investigate the underlying mechanism, we analyzed the expression dynamics of Foxp3, a master transcription factor for Tregs, in peripheral blood mononuclear cells. We found that Foxp3 expression levels closely mirrored the abundance of CD4⁺CD25⁺ T cells (Fig. 3H), suggesting a central role for Foxp3 in CD4⁺CD25⁺ T cell-mediated immunoregulation.

To elucidate the transcriptional role of Foxp3 during influenza infection, we quantified several key factors in the FoxO signaling pathway in chicken peripheral blood mononuclear cells (PBMCs) at 5 dpi on the basis of our previous analysis of potential chFoxp3 target genes and pathways (Fig. 2F). The results revealed significant upregulation of several genes within this pathway, including S1PR1, S1PR4, IL-7R, FoxO3, FoxO4, BCL6, SIRT1, KLF2, and EP300, with IL-10 showing the most pronounced increase (Fig. 3I). Elevated serum IL-10 levels further support its functional importance during infection (Fig. 3H). IL-10 serves as a central immunosuppressive cytokine that inhibits proinflammatory cytokine production and preserves immune balance [42]. Nevertheless, the precise mode of action and molecular mechanism whereby avian Foxp3 controls IL-10 expression requires further investigation. To elucidate the transcriptional role of Foxp3 during influenza infection, we quantified several key factors in the FoxO signaling pathway in chicken peripheral blood mononuclear cells (PBMCs) at 5 dpi on the basis of previously identified potential Foxp3 target genes and pathways (Fig. 2F). The results revealed significant upregulation of these putative Foxp3 target genes, including S1PR1, S1PR4, IL-7R, FoxO3, FoxO4, BCL6, SIRT1, KLF2, and EP300, most notably IL-10 (Fig. 3I). Elevated serum IL-10 levels further support its functional importance during infection (Fig. 3H). IL-10 serves as a central immunosuppressive cytokine that inhibits proinflammatory cytokine production and preserves immune balance [42]. Nevertheless, the precise mode of action and molecular mechanism whereby avian Foxp3 controls IL-10 expression requires further investigation.

These findings collectively demonstrate that chFoxp3 plays a pivotal regulatory role throughout cellular processes. The mechanism by which it regulates gene transcription through binding to T3G motifs not only elucidates the uniqueness of the avian transcriptional reprogramming system but also provides critical guidance for subsequent research.

### ChFoxp3 directly binds to the T3G motif within the IL-10 promoter and specifically activates the transcription of chIL-10

To establish a direct link between IL-10 transcriptional regulation and Foxp3 binding to the T3G motif, we first defined the genomic context of IL-10 through genome-wide scanning. The gene is located on chromosome 26, and its open reading frame is composed of five exons (Fig. 3J). Notably, a T3G motif was identified at position −403 bp upstream of the IL-10 transcription start site, suggesting a potential Foxp3 binding site. To test this hypothesis, we synthesized a biotinylated oligonucleotide probe encompassing this motif for EMSA. The results demonstrated that chFoxp3 specifically bound to this probe in a concentration-dependent manner (Fig. 3K), confirming that chFoxp3 directly binds the IL-10 promoter to mediate its transcriptional activation.

**Fig. 4:**
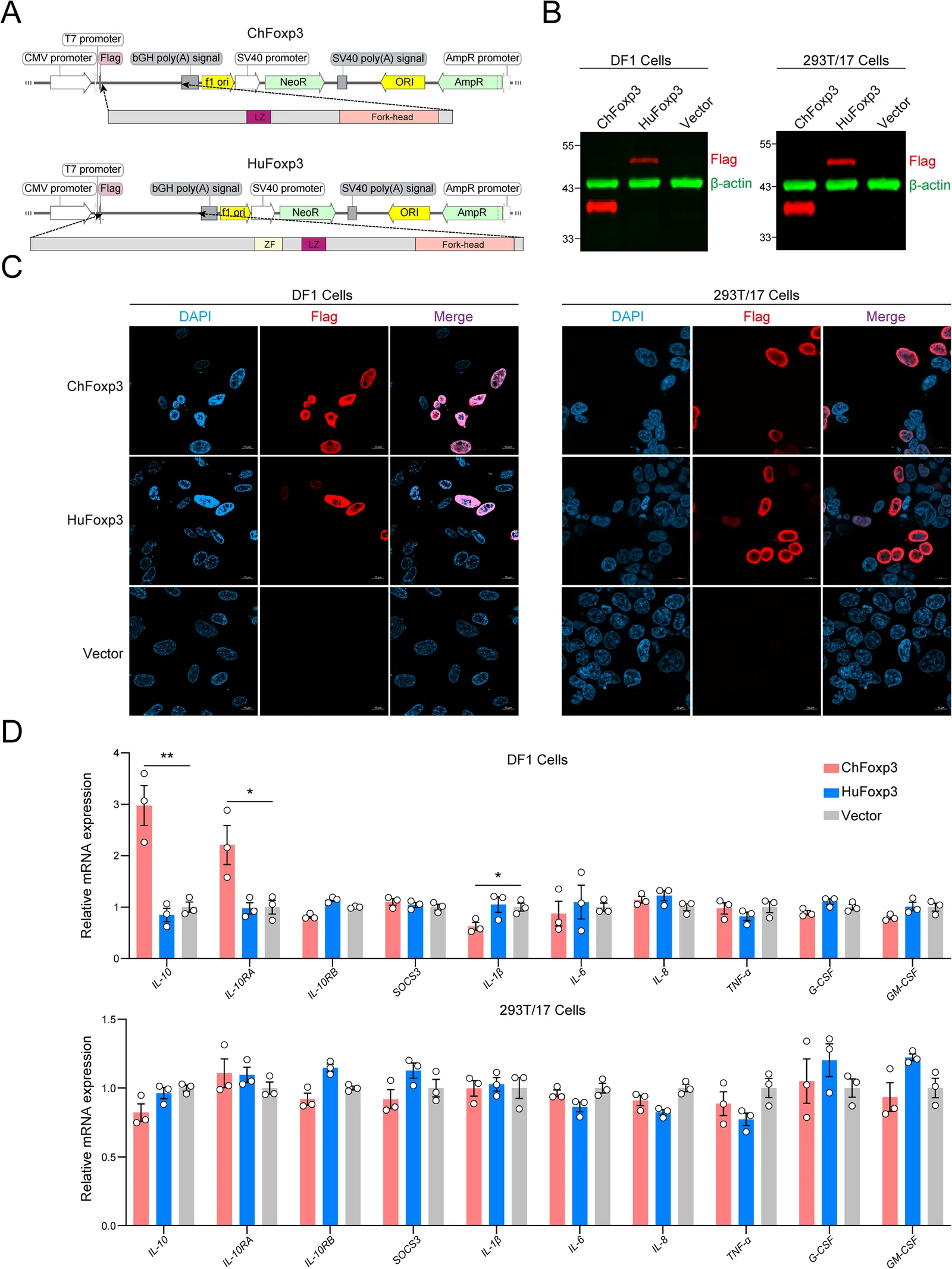
ChFoxp3 promotes the transcription of IL-10. **A** Schematic diagram of chFoxp3 and huFoxp3 plasmid construction. **B** Western blot validation of chFoxp3 and huFoxp3 expression in 293 T/17 and DF-1 cells. Red and green represent the Flag and β-actin bands, respectively. **C** Subcellular localization of chFoxp3 and huFoxp3 in 293 T/17 and DF-1 cells. The red and blue staining represent the Flag tag fusion Foxp3 protein and the nucleus, respectively. **D** Relative mRNA expression of inflammation-related cytokines (IL-10, IL-10RA, IL-10RB, SOCS3, IL-1β, IL-6, IL-8, TNF-α, G-CSF, and GM-CSF) in chFoxp3- and huFoxp3-overexpressing DF-1 and 293 T/17 cells (n = 6). *, 0.01 < *P* < 0.05; **, 0.001 < *P* ≤ 0.01; ***, *P* ≤ 0.001

To further investigate the transcriptional regulatory function of Foxp3 at the cellular level, we constructed recombinant eukaryotic plasmids encoding full-length chicken or huFoxp3 (Fig. 4A). Protein expression analysis confirmed that both proteins were successfully expressed in mammalian 293 T/17 cells and chicken embryo fibroblast DF-1 cells (Fig. 4B). Laser confocal microscopy revealed that both chFoxp3 and huFoxp3 were localized in the nucleus (Fig. 4C), which is consistent with the nuclear localization of their transcription factors. Further experiments revealed that the expression of IL-10 was significantly upregulated in DF-1 cells overexpressing chFoxp3, along with the upregulation of the IL-10RA receptor. Moreover, the proinflammatory cytokine IL-1β was downregulated (Fig. 4D). In contrast, the overexpression of chFoxp3 or huFoxp3 in 293 T/17 cells did not induce changes in the expression of inflammatory-related cytokines, demonstrating that chFoxp3 may regulate inflammatory responses by increasing IL-10 transcription in chickens. Collectively, our findings support a model in which chFoxp3 operates via species-specific upregulation of avian IL-10 to temper inflammatory responses, a mechanism that does not generalize to the human ortholog in standard cell models.

### ChFoxp3 directly drives the epigenetic activation of chIL-10 in a T3G-dependent manner

**Fig. 5:**
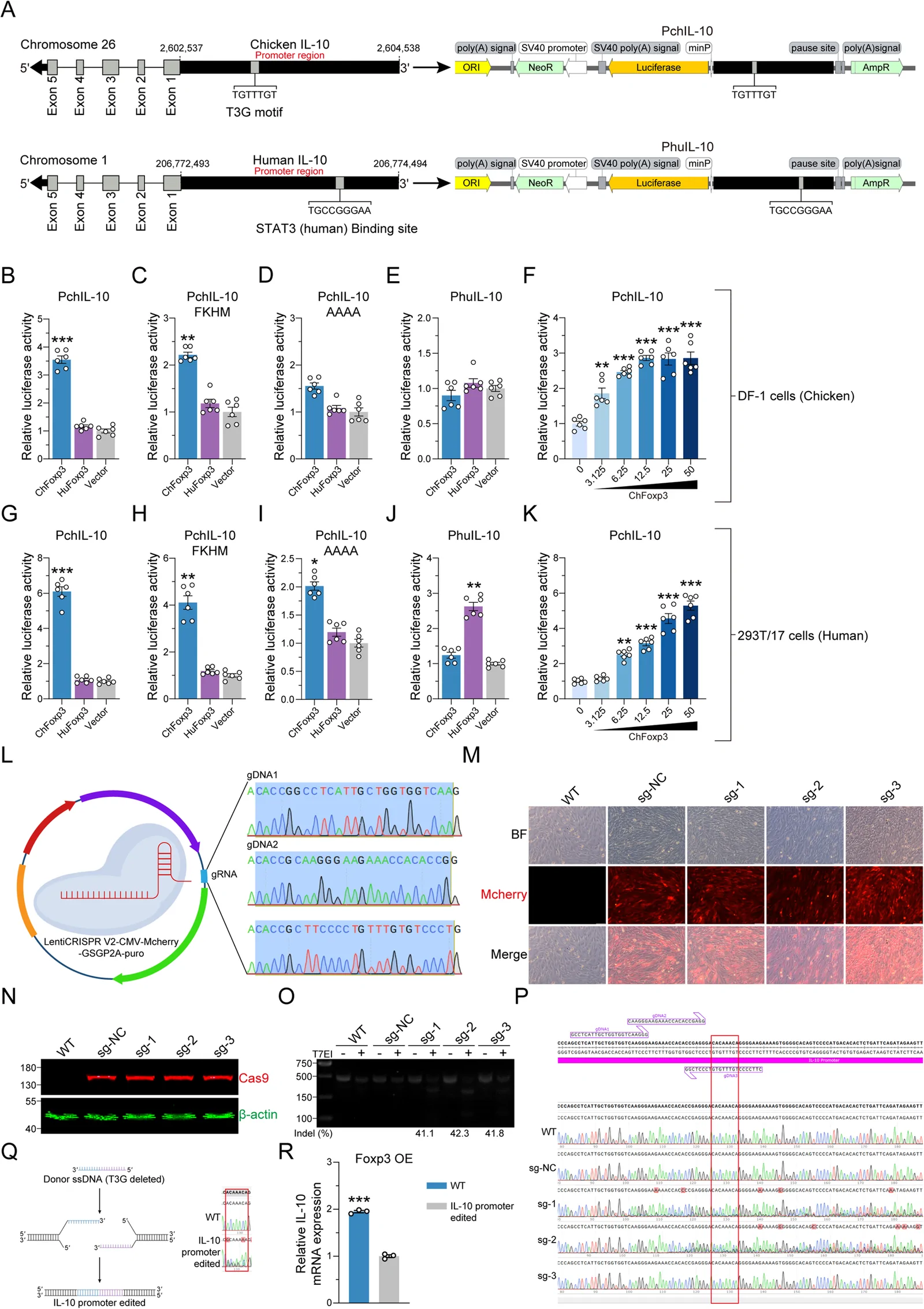
ChFoxp3 activates the IL-10 promoter. A Schematic representation of the IL-10 promoter region in chickens and humans, illustrating chromosomal localization, gene structure, and the design of reporter gene constructs. The plasmid PchIL-10 contains a T3G motif (TGTTTGT), and the plasmid PhuIL-10 contains a known human STAT3 binding motif (TGCCGGGAA). B-F Relative activity of IL-10 promotors in DF-1 cells (n=6). G-K Relative activity of IL-10 promotors in 293T/17 cells (n=6). L Illustration and sequencing results of the pLentiCRISPR v2-cmv-mCherry-GSGP2A-puro plasmids. M Verification of mCherry expression in cells. N Western blot results showing Cas9 expression in cells. Red and green represent the Cas9 and β-actin bands, respectively. O T7EI assay results for genome disruptions. P Sanger sequence results for genome disruptions. Q Sanger sequence results for IL-10 promoter edited DF-1 cells. R Relative IL-10 mRNA expression in chFoxp3 overexpression cells. OE: over expression

To elucidate the transcriptional regulatory effect of chFoxp3 on IL-10 between avians and mammals, we characterized and compared the chromosomal localization, exon organization, and promoter architecture of the IL-10 gene in chickens and humans. The promoter architectures of human and chicken IL-10 differ significantly: the human promoter contains a characteristic STAT3-binding site, whereas the chicken promoter lacks this element and instead possesses a T3G motif, suggesting divergent regulatory mechanisms (Fig. 5A). Previous studies have demonstrated that the human transcription factor STAT3 induces IL-10 gene expression by binding to a consensus sequence (TGCCGGGAA) at position −120 of the IL-10 promoter [43]. Although huFoxp3 lacks direct binding affinity for the IL-10 promoter, it functions as a co-transcription factor that cooperatively enhances STAT3-mediated IL-10 transcription [44]. To further assess the transactivation capacity of Foxp3 from both species on the IL-10 promoter, we cloned a genomic fragment encompassing the region immediately upstream of the chIL-10 or huIL-10 transcription start site into the luciferase reporter plasmid pGL6 TA (Fig. 5A).

In cells co-transfected with the reporter plasmid and the Foxp3 expression plasmid, chFoxp3 strongly activated the chicken IL-10 promoter, whereas huFoxp3 did not activate the chIL-10 promoter. Notably, huFoxp3 did not activate the huIL-10 promoter in chicken cells, but it did activate the human IL-10 promoter in human cells, which may be related to its co-transcription factors. These results indicate that the transcriptional activation function of Foxp3 is species-specific, reflecting evolutionary differences in the immune system. To assess the functional significance of the T3G motif, we generated mutant chIL-10 promoter reporter constructs in which the T3G motif was replaced with either an FKHM sequence or a poly(A) sequence (Fig. 5A). Compared with the wild-type promoter, mutation of the T3G motif significantly impaired the ability of chFoxp3 to activate the reporter gene (Fig. 5B-K). These results indicate that the T3G motif in the regulatory element of the target gene is essential for the transcriptional activation function of chFoxp3. In addition, by adjusting the transfection dose of the chFoxp3 expression plasmid, we found that activation of the chIL-10 promoter by chFoxp3 was dose dependent (Fig. 5B-K). To prioritize candidate target genes for validation, we applied three selection criteria: (i) presence of at least one T3G repeat unit (TGTTTGT) in the promoter region (2,000 bp upstream of the transcription start site) based on genome-wide scanning; (ii) cross‑species intersection with mammalian Foxp3 target genes curated from seven public databases (TRRUST, FIMO_JASPAR, CHEA, GTRD, ChIP_Atlas, PWMEnrich_JASPAR, and hTFtarget); and (iii) biological relevance to immune regulation, particularly in Treg‑mediated immunosuppression and inflammatory responses. Based on these criteria, we selected FoxO3, S1PR1, and IL7R for reporter assays. The results showed that chFoxp3 activated the FoxO3 promoter but did not activate the S1PR1 or IL-7R promoters under the conditions tested (Fig. S5). Three sgRNAs targeting the T3G motif in the chicken IL-10 promoter region were designed and synthesized, and delivered together with Cas9 into DF-1 cells via lentiviral transduction. Subsequently, site-specific deletion of the T3G motif was also performed using ssDNA. By overexpressing chFoxp3 in partially edited cells, we found that deletion of the T3G motif in the chicken IL-10 promoter weakened the transcriptional activation effect of chFoxp3 compared with that in wild-type cells (5L-R).

### Key residues in chFoxp3 define the structural basis for species-specific recognition of the T3G motif

**Fig. 6:**
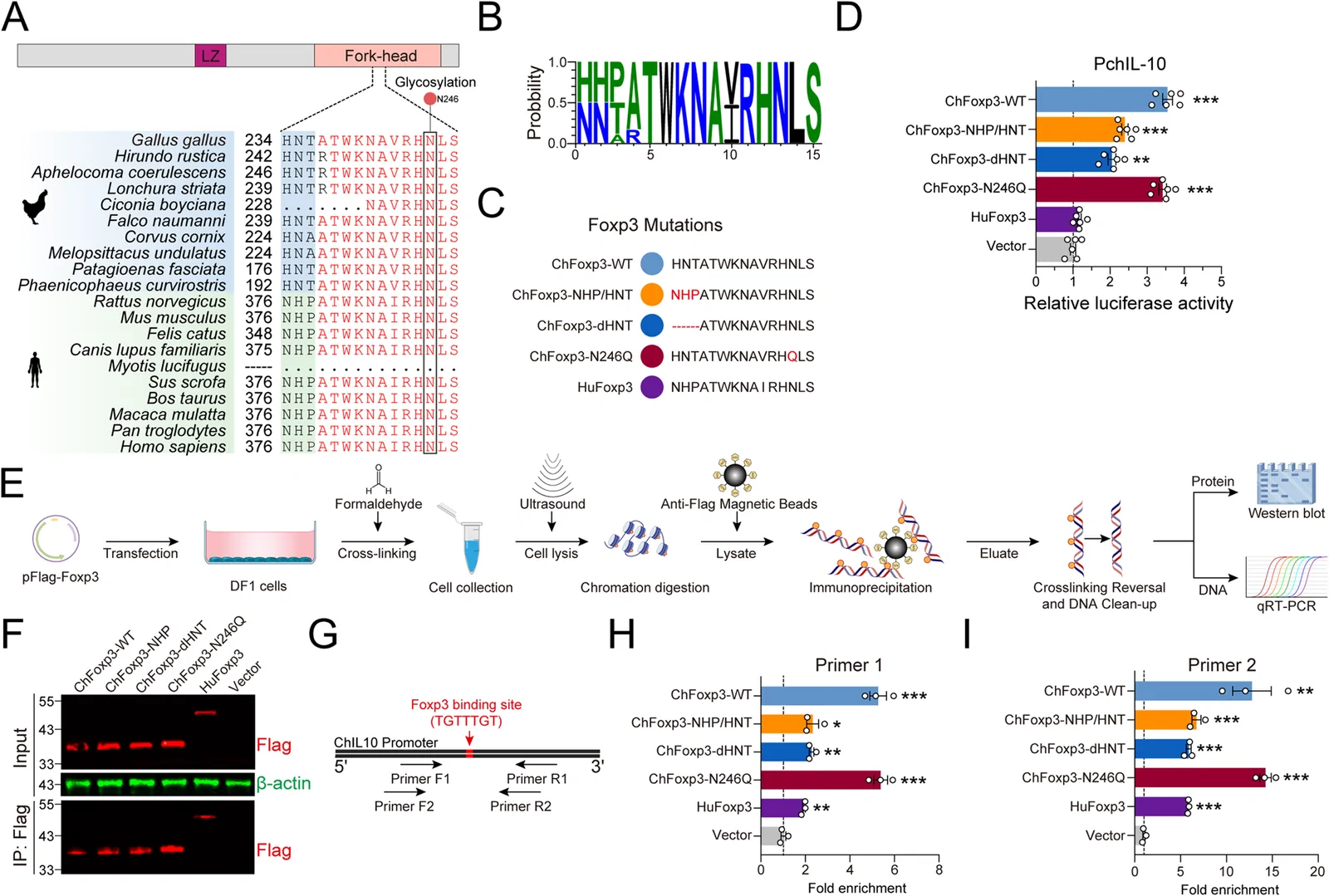
ChFoxp3 interacts with the T3G motif in the IL-10 promoter region via its Forkhead domain. **A** Alignment of a segment of the Foxp3 Forkhead domain. Fully conserved amino acids are highlighted in shaded red. **B** Sequence logo representation of a segment of the Foxp3 Fork-head domain. **C** Schematic representation of the constructed Foxp3 mutant plasmid. **D** Relative activity of PchIL-10 in Foxp3-overexpressing cells (n = 6). **E** Schematic workflow of the ChIP assay. **F** Foxp3 was expressed in DF-1 cells, and the lysate was incubated with anti-Flag magnetic beads and then analyzed via Western blotting. **G** Schematic of primer design for ChIP-qRT-PCR. **H-I** Relative amount of DNA bound by different Foxp3 types under two distinct primer sets (n = 3). *, 0.01 < *P* < 0.05; **, 0.001 < *P* ≤ 0.01; ***, *P* ≤ 0.001

Next, we focused on the fork-head domain to elucidate the mechanism underlying their functional divergence. Comparative analysis of the fork-head domain sequences between avian species (represented by chicken) and mammalian species (represented by human) Foxp3 revealed that avian Foxp3 fork-head domains consistently contain a specific HNT (234–236 aa) motif. In contrast, the corresponding region in mammalian sequences is the NHP region (376–378 aa). These flexible amino acids are situated between two α-helices of the fork-head domain, and their variation may substantially alter the spatial configuration of the protein (Fig. 6A-B; Fig. S2). Additionally, Foxp3 from all species contains a conserved N-glycosylation site (N246) in the Fork-head domain. Studies indicate that glycosylation may significantly influence protein function, particularly in protein interactions or molecular binding (Fig. 6A-B; Fig. S2) [45].

To investigate whether these sites affect avian Foxp3 functionality, we introduced site-directed mutations or deletions at the targeted amino acid residues to generate chFoxp3 mutants. These mutants, along with wild-type chFoxp3, were designated WT, NHP/HNT, dHNT, and N246Q, respectively (Fig. 6C). Co-transfection with the chIL-10 promoter reporter plasmid demonstrated that mutating or deleting the HNT motif significantly attenuated chFoxp3-mediated activation of the chIL-10 promoter. In contrast, alteration of the glycosylation site (N246Q) had no significant effect on chFoxp3 function to activate chIL-10 transcription (Fig. 6D). Various Foxp3 expression plasmids were transfected into DF-1 cells for ChIP-RT-qPCR analysis (Fig. 6E). The results demonstrated that chFoxp3 specifically binds to the T3G motif in the IL-10 promoter. This binding was attenuated when the HNT sequence of chFoxp3 was altered or deleted. huFoxp3 also bound to the T3G motif in the chIL-10 promoter, although with reduced affinity compared with that of chFoxp3 (Fig. 6F-I).

To provide structural support for this conclusion, we used AlphaFold3 to generate models of chFoxp3 and huFoxp3 bound to T3G, and Supplementary Fig. S6 was used to visually illustrate the positional differences between the HNT and NHP motifs in three-dimensional space (Fig. S7A). Further simulation results showed that the T236P mutation in chFoxp3 led to the loss of one hydrogen bond between T236 and L240, thereby causing structural changes, which may underlie its functional effects (Fig. S7B).

**Fig. 7:**
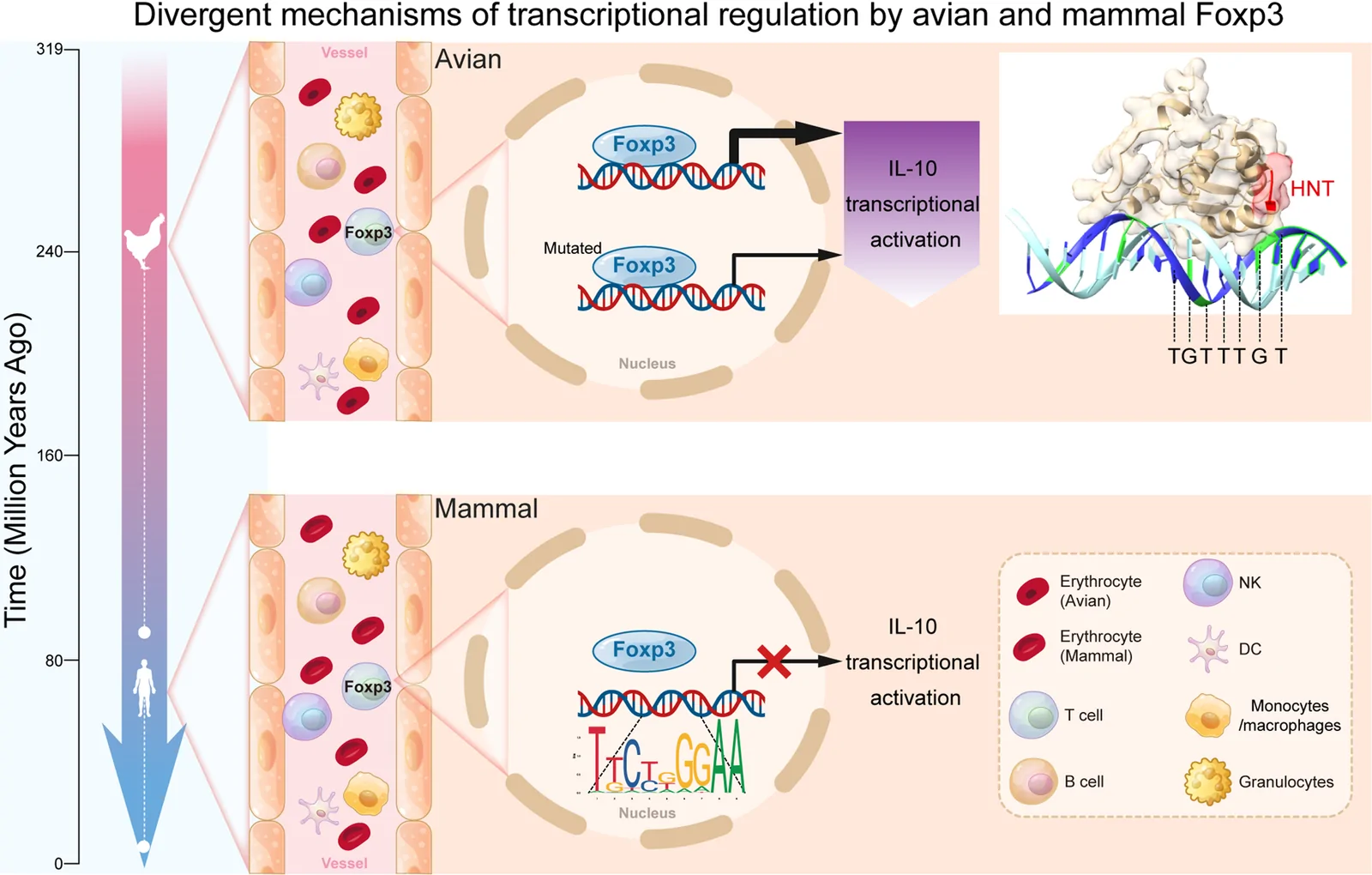
Schematic diagram illustrating the divergent mechanisms of transcriptional regulation by avian and mammalian Foxp3. Foxp3 was markedly upregulated in the H9N2 AIV chicken infection model. Mechanistically, avian Foxp3 can directly bind to the T3G motif within the IL-10 promoter region, thereby epigenetically activating IL-10 transcription and modulating the host immune inflammatory response. In contrast, mammalian Foxp3 lacks this ability, highlighting a fundamental functional difference that underscores the divergent evolutionary trajectories of immune regulation across species. In conclusion, our findings elucidate a novel immune regulatory pathway, providing a theoretical foundation for further understanding the evolutionary differences in regulation between avian and mammalian species

Overall, in a chicken model of H9N2 infection, the upregulated transcription factor chFoxp3 binds to the T3G motif within regulatory elements of specific genes, thereby mediating epigenetic modifications of its target genes (Fig. 7). On the basis of these findings, we propose a novel molecular mechanism for the transcriptional regulation of IL-10 by chFoxp3. Under specific physiological or pathological conditions, chFoxp3 directly binds to the T3G motif within its promoter to activate target genes such as IL-10, whereas human/mammalian Foxp3 lacks this capacity. This fundamental functional divergence underscores distinct evolutionary paths in immune regulation across species. Our study elucidates a previously unrecognized avian immunoregulatory pathway that may serve as a critical hub for mitigating virus-induced hyperinflammation and maintaining immune homeostasis.

## Discussion

Chickens, as significant economic species, serve as excellent models for comparative immunology research because of their high-density farming conditions and increased exposure to pathogens. However, a comprehensive understanding of avian immune cell functions, particularly immune tolerance and regulation, is lacking. Although birds possess a group of cells similar to mammalian Treg cells, their phenotype and function are not well understood. Only recently was the signature transcription factor Foxp3 of Treg cells characterized [12]. Questions remain concerning whether chFoxp3 functions as a typical transcription factor similar to its mammalian counterpart, the DNA motif it preferentially binds to, its conservation with mammals, and the downstream target genes it directly regulates. The ability of these genes to mediate chicken Treg cell function is still unknown.

In this study, bioinformatics analysis and cloning of chFoxp3 revealed significant differences from mammalian Foxp3 molecules. chFoxp3 lacks the conserved DNA-binding ZF domain found in mammalian Foxp3 but retains the hallmark Fork-head domain of the Fox protein family. Structural modeling further highlighted the overall structural differences between chFoxp3 and its mouse homolog, despite their nearly identical Fork-head domain folding. The absence of the ZF domain, which promotes DNA binding in mammals, raises questions about its functional impact on the DNA-binding ability of chFoxp3. To address this, the DNA-binding ability of full-length chFoxp3 was expressed, purified, and tested. Electrophoretic mobility shift assays revealed that chFoxp3 does not bind to a single FKHM structure or its reverse complementary variant but specifically interacts with the T3G microsatellite sequence. These findings suggest that chFoxp3 may regulate gene expression by recognizing microsatellite sequences.

Through genome-wide screening of T3G structures in the chicken genome combined with cross-species interaction analysis of human Foxp3 target genes, we identified 2,231 potential chFoxp3 target genes. KEGG pathway enrichment analysis revealed that the FoxO signaling pathway was the most significantly enriched biological process. Moreover, IL-10 exerts immunosuppressive effects by inhibiting proinflammatory cytokine production and limiting helper T cell activity.

We established an H9N2 AIV infection model in chickens and used flow cytometry to analyze the dynamic changes in the proportions of various T cell subsets during infection. These results suggest that H9N2 AIV induces immune dysregulation in avian hosts, particularly highlighting the characteristics of Foxp3, a key transcription factor that regulates Treg cell function. Notably, chickens infected with H9N2 AIV presented a transient increase in CD4⁺CD25⁺ T cell proportions at 5 dpi, which returned to baseline levels by 7 dpi. The current view is that host mortality in influenza infections is attributed primarily to cytokine storm syndrome. Although H9N2 AIV typically causes subclinical infections in chickens as a low pathogenic strain, clinical deterioration due to coinfections and secondary complications has been documented, although the underlying mechanisms remain unclear [46]. Our study may help fill the knowledge gap regarding H9N2-mediated disruption of immune homeostasis, particularly its impact on CD4⁺CD25⁺ T cell-mediated immune tolerance. The changes in the expression patterns of these immune regulatory factors during H9N2 AIV infection suggest direct or indirect chFoxp3 mediation, pointing to a potential novel transcriptional regulatory mechanism involved in avian immune responses.

In vitro cell experiments further demonstrated that chFoxp3 can act as a transcription factor to promote IL-10 expression. Mechanistically, chFoxp3 was observed to directly bind to the T3G motif at the IL-10 promoter location in the genome, thereby transactivating IL-10 transcription, which appears to be fundamentally different from that of mammalian Foxp3. Moreover, the conserved HNT sequence in avian Foxp3 and the conserved NHP sequence at the same position in mammals may represent key amino acid sites contributing to this interspecies difference. Previous studies have shown that the human transcription factor STAT3 can induce IL-10 gene expression by binding to the conserved sequence (TGCCGGGAA) at the -120 position of the IL-10 promoter [43]. Although human Foxp3 itself lacks direct binding affinity to the IL-10 promoter, it can act as an auxiliary transcription factor to synergistically increase STAT3-mediated IL-10 transcription [44]. Compared with the mammalian conserved "Foxp3-STAT3-IL-10" indirect regulatory model, the autonomous direct regulatory ability of chFoxp3 suggests an evolutionary divergence in immune regulatory pathways. These findings point to a potential new mechanism of avian immune balance, where this pathway might serve as a key hub for birds to counteract excessive inflammation caused by viral infections and maintain immune homeostasis. The direct positive regulation of the anti-inflammatory factor IL-10 by Foxp3 offers an expanded perspective on the avian immunosuppressive network. This study provides new insights into avian-specific immune regulatory mechanisms while offering a valuable framework for understanding nonmammalian immune regulation. It is also important to note that the absence of transcriptional activation in our reporter assays does not exclude the possibility that chFoxp3 may repress certain target genes, such as S1PR1 or IL-7R, through indirect mechanisms or under different cellular contexts (Fig. S5). Foxp3 is known to function as both a transcriptional activator and a repressor, depending on its interaction with cofactors and the chromatin environment. The main limitation of this study is that, as a newly identified transcription factor, it remains unclear whether chFoxp3 binds to additional motifs. In the future, the development of chFoxp3-specific antibodies and the application of other techniques may help identify additional target genes or cofactors of chFoxp3. However, this does not conflict with the conclusions of the present study; rather, these findings will collectively help elucidate the gene regulatory network of chFoxp3. Despite these limitations, integrating validated specific DNA motifs with genome-wide sequence scanning remains an effective strategy for generating biologically meaningful hypotheses and guiding subsequent functional validation.

## Supplementary Information

Supplementary Material 1.

Supplementary Material 2.

## Data Availability

All relevant data are available in the article or in the accompanying Supplementary Information file.

## Abbreviations

Foxp3: Forkhead box P3
Treg: Regulatory T cell
IL: Interleukin
AIV: Avian influenza virus
TGF-β: Transforming growth factor beta
CTLA-4: Cytotoxic T-lymphocyte associated protein 4
IFN: Interferon
SPF: Specific-pathogen-free
FBS: Fetal bovine serum
Dpi: Days post-infection
H&E: Hematoxylin and eosin
BSA: Bovine serum albumin
PFA: Paraformaldehyde
PBMC: Peripheral blood mononuclear cell
STAT3: Signal transducer and activator of transcription 3
IL-7R: Interleukin-7 receptor
G-CSF: Granulocyte colony-stimulating factor
GM-CSF: Granulocyte-macrophage colony-stimulating factor
SOCS3: Suppressor of cytokine signaling 3
TNF-α: Tumor necrosis factor alpha
Th1: T helper 1
Th17: T helper 17
