# Supplementary material for: Avian Foxp3 acts as a switch for IL-10 by directly binding the T3G motif: revealing a novel epigenetic modulation paradigm
Source: Cell Commun Signal. 2026 Jun 25;24:493. doi: 10.1186/s12964-026-02967-0 (PMC13584488; doi:10.1186/s12964-026-02967-0)
Supplement: Supplementary file 1 [file 12964_2026_2967_MOESM1_ESM.docx]

**Fig. S1** Amplification and alignment of chFoxp3. **A** Amplification of chFoxp3. M, DNA ladder marker (2,000 bp). **B** Alignment of protein sequence of Foxp3. Fully conserved amino acids are highlighted in shaded red, while partially conserved amino acids are enclosed within blue boxes. NLS, Nuclear Localization Signal.

**Fig. S2** Comparative structural characterization of chFoxp3 and mouse Foxp3. Light green indicates the proline-rich domain, grayish pink indicates the leucine zipper domain, light blue indicates the fork-head domain, rust orange indicates the ZF domain, steel blue indicates the RUNX1-binding domain.

**Fig. S3** Recombinant chFoxp3 protein expression. **A** Hydrophobicity analysis of the chFoxp3 sequence. The x-axis represents the amino acid positions, and the y-axis indicates the hydrophobicity score. **B** Disorder analysis of the chFoxp3 sequence. The x-axis represents amino acid positions, and the y-axis shows the probability of disorder. **C** ChFoxp3 expressed at varying temperatures (15℃-37℃). **D** Purification profile of chFoxp3 eluted with imidazole gradients (20-500 mM). **E** Native PAGE of purified recombinant chFoxp3 under non-denaturing conditions. **F** SEC-HPLC elution profile of recombinant chFoxp3. The chromatogram shows two major peaks corresponding to the dimeric and monomeric forms. **G** Native-PAGE analysis of the two major peaks collected from SEC-HPLC. **H** SDS-PAGE analysis of the two major peaks collected from SEC-HPLC. **I** Far-UV circular dichroism (CD) spectrum of recombinant chFoxp3. The spectrum was recorded in the wavelength range of 180-260 nm in PBS. The mean residue ellipticity (θ) is expressed as millidegrees (mdeg).

**Fig.** **S4** Enrichment analysis of chFoxp3 target genes. **A** Enrichment of the chFoxp3 target genes in KEGG pathway annotation. The x-axis shows the numbers of genes enriched in each pathway (y-axis). **B** Enrichment of the chFoxp3 target genes in GO items. The x-axis shows the numbers of genes enriched in each item (y-axis). The BP, MF, CC denote the biological process, molecular function and cellular component, respectively. **C** Top 20 significant enriched GO items of the chFoxp3 target genes. The x-axis shows enrichment factor of each GO item (y-axis). The size of the spheres reflects the number of genes (labelled beside the balls) enriched in each item. The color of the spheres demonstrates the significance of the enrichment analysis.

**Fig. S5** Functional testing of candidate chFoxp3 target promoters identified by T3G motif scanning. **A** Schematic representation of the promoter regions of FoxO3, S1PR1, and IL7R in chicken and human genomes, including chromosomal localization, gene structure, and reporter construct design. The reporter plasmids (PchFoxO3, PchS1PR1, and PchIL7R) each contain at least one T3G repeat unit (TGTTTGT). **B** Relative luciferase activity of FoxO3, S1PR1, and IL7R promoter reporters in 293T/17 cells (n = 6).

**Fig. S6** Effects of the HNT site mutation on the structure of Foxp3. **A** Structural representation of Foxp3 (*Gallus gallus* and *Homo sapiens*) binding to T3G microsatellites. **B** Structural changes caused by the HNT-to-NHP mutation in Foxp3 (*Gallus gallus*).

**Supplementary Table information**

**Table S1.** Genome-wide localization of the T3G motif across gene promoters in the chicken genome.

**Table S2.** Public database-annotated target genes of huFoxp3.

**Table S3.** Intersecting target genes shared between huFoxp3 and chFoxp3

**Table S4.** KEGG enrichment analysis of chFoxp3 target genes.

**Table S5.** GO enrichment analysis of chFoxp3 target genes.

**Table S6.** Primer sequences.

**Table S7.** dsDNA sequences for EMSA.

**Table S8.** sgDNA and ssODN sequences.
